# Predicting Colorectal Cancer Using Residual Deep Learning with Nursing Care

**DOI:** 10.1155/2022/7996195

**Published:** 2022-02-27

**Authors:** Lina Wang

**Affiliations:** The Third Affiliated Hospital of Qiqihar Medical College, Qiqihar, Heilongjiang 161000, China

## Abstract

Presently, colorectal cancer is the second most dangerous cancer; around 13% of people have been affected; and it requires an effective image analysis and earlier cancer prediction (IAECP) system for reducing the mortality rate. Here, the IAECP system uses MRI radio imaging for predicting colorectal cancer. During this process, high- and low-level features are required to examine cancer in an earlier stage. Due to the limitation of the conventional feature extraction process, both features are difficult to extract from cancer suffered locations. Hence, a deep learning system (DLS) is used to examine the entire bowel MRI image to identify the cancer-affected location, feature extraction, and feature training process. Furthermore, the DLS-based IAECP system helps improve the overall colorectal cancer identification accuracy for further process. The derived bowel features are trained by applying the residual convolution network, which minimizes the error between predicted and actual values. Finally, the test query images are compared with the trained image by applying the sum, which is more absolute to the cross-correlation template feature matching (SACC) algorithm. The experimental process is performed using 100,000 histological data sets, which is considered a publicly available data set. Moreover, the introduced method does not use generic features, whereas the deep learning features help improve the overall IAECP prediction rate (99.8%) ratio as predicted at lab-scale analysis.

## 1. Introduction

Worldwide, colorectal cancer [1] is considered one of the common cancers. In 2012, around 1,360,000 cases were suffered due to colorectal cancer, among 447,000 patients in Europe [2]. This cancer is the second most common cancer; due to this rectal cancer, 125,000 people have died. The environmental influence, hereditary changes, and genetic mutation accumulations were the main rectal cancer risk factors [3]. Around 80% of rectal cancer growth starts at the colon's inner lining or rectum, named the polyps. These polyps [4] are changed into cancer over time, depending on two types: adenomas (adenomatous polyps) and inflammatory polyps. Here, the adenomatous polyps are sometimes converted to cancer that is a precancerous condition. The inflammatory polyps are common that are not precancerous in nature. This colorectal cancer has a risk-based polyp with larger than 1 cm, and more than two polyps found in the colon. After removing the polyp from the colon, most of the time, dysplasia is formed based on the precancerous condition, whereas it is more unique than the cancer cell. This colorectal cancer is categorized into several types [5], such as adenocarcinoma (it is the most common, developed in the large intestine and mucus-producing glands), gastrointestinal carcinoid tumor (developed in large intestine hormone-producing cells), gastrointestinal stromal tumor (developed in Cajal interstitial cells), primary colorectal lymphoma (developed in the colon), and sarcoma (developed in blood vessels). This colon or colorectal cancer has several symptoms [6], such as diarrhea, bleeding in stool, fatigue, unexpected weight loss, and abdominal discomfort. Based on the condition, the main risk factors [7] are inherited syndromes, family history, sedentary lifestyle, unhealthy diet, obesity, and smoking. Due to these risk factors, colorectal cancers occur in people. Hence, the normal screening process [8] should be taken around the age of 50. Most of the time, the manual screening process gives false results, creating serious issues called death. To overcome these serious issues, a computer-aided automatic system [9] has to be created and predicts colorectal cancer without any difficulties or complexity. The automatic detection system uses various machine learning and image analysis techniques [10] to predict the changes in their colon. Several methods such as support vector machine, neural networks, convolution networks, extreme learning approach, feedforward network, region segmentation, edge analysis, and dual clustering approaches are used to examine the colorectal MRI image. Most of the automatic system uses MRI image instead of CT image because it provides detailed information regarding the human body. The in-depth information helps predict the exact changes or modulation in their function. With the help of the discussion, several researchers are predicting colorectal cancer using machine learning techniques. A deep learning system (DLS) is utilized to analyze the entire bowel MRI image to determine the cancer-affected location, feature extraction, and feature training process. Moreover, the DLS-based image analysis and earlier cancer prediction (IAECP) system help enhance the total colorectal cancer recognition accuracy for further processes. The derived bowel features are trained by employing the residual convolution network, which reduces the error between forecasted and real values. Lastly, the test query images are compared with the trained image by applying the sum, which is more absolute to the cross-correlation template feature matching (SACC) algorithm.

## 2. Related Works

Here, few researchers' opinions are analyzed to get the knowledge regarding the colorectal cancer identification process. Ahmed et al. [11] predicted the survival rate of colon-cancer-affected patients using an artificial neural network. During the analysis process, the three-layer of the feedforward neural network is used to examine the survival rate. Initially, the information of colon-infected people is gathered and processed using a nonlinear regression device called an artificial network. According to their risk factor, the network computes the survival rate, where the cancer stage and seriousness have created system efficiency more liable when compared with the clinic pathology approaches. Thus, the artificial neural network ensures 86.9% of efficiency.

Kather et al. [12] analyzed colorectal cancer-affected survival rate using deep convolution networks. During this process, 100,000 histological images are collected from patients who are in tissue slides. The collected tissue slides are decomposed into subimages that are examined in terms of providing deep stroma scores. This score value ensures the prognostic factor and overall survival rate of the patient. The created system is evaluated using the 409 patient colorectal details collected from multiple institutions in Germany. From the analysis, the system ensures up to 95.34% accuracy while predicting the exact survival rate of colorectal cancer-affected patients.

Iizuka et al. [13] created an effective gastric and colonic epithelial cancer detection system using convolution and recurrent neural network. During this process, in this work, colorectal cancer histological images are collected from patients, and effective features are extracted. The extracted features are trained by the convolution network and recurrent neural network. The introduced network examines the whole slide images and categorizes adenoma, adenocarcinoma, and non-neoplastic tumors. The system's efficiency is evaluated using the experimental results in which the system predicts the colon tumor up to 0.97 of the area under curve value.

Daniel et al. [14] developed the backpropagation neural network-based automatic computer-aided system to predict the breathomics gastric cancer. During this process, 49 gastric cancer patients and 30 gastric ulcer patient information are collected by the initial screening process. The collected images are processed by the backpropagation approach, which classifies the patient information by calculating the ethyl acetate, 2-propanol, carbon disulfide, acetone, ethyl alcohol, and other stomach-related issues. Based on the criteria, patients are classified into positive case, suspected, and normal. The system's efficiency is evaluated using experimental results in which the system recognizes gastric cancer with up to 96.45% accuracy based on the various author's opinions. The artificial intelligent technique called a neural network played a vital role in predicting colorectal cancer cancers. In this work, an effective ResNet deep neural network [15] is used for classifying colorectal cancer by considering their opinion. In addition to this, the created system utilizes the convolution network to extract the features based on the traditional techniques, failing to extract high-level information from the colorectal image. So this network derives the high- and low-level features that are mostly helping recognize the colorectal images with maximum accuracy. Zuo et al. [16] suggested the univariate and multivariate Cox regression analyses for predicting the prognosis of colorectal cancer. The Cancer Genome Atlas (CAA) data source was used to evaluate modification of colorectal cancer (CRC) prognosis in gene expression profiles and clinicopathological data of patients of colon adenocarcinoma (COAD) and rectum adenocarcinoma (READ) expression. Between COAD/READ and standard tissue samples, differentially expressed mRNAs (DEMs) were found. An mRNA panel signature was created using univariate and multivariate Cox regression analyses to predict the overall survival (OS) in patients with CRC. Ooft et al. [17] discussed the prospective clinical study, and they demonstrate the feasibility of producing and testing patient-derived tumor organoids (PDOs) to evaluate sensitivity to chemotherapy. Their patient-derived tumor organoids test forecasted the response of the biopsied lesion in more than 80% of patients preserved with irinotecan-based therapy without miscategorizing patients who would have helped from treatment. However, this association was particular to irinotecan-based chemotherapy, and the patient-derived tumor organoids failed to forecast outcomes for treatment with 5-fluorouracil plus oxaliplatin.

Then the rest of the manuscript is arranged as follows: Section 2 deals with the IAECP-based colorectal cancer prediction process, Section 3 assesses the efficiency of the IAECP system, and Section 4 provides the conclusion of the study.

## 3. Image Analysis and Earlier Cancer Prediction (IAECP) System

**Figure 1:**
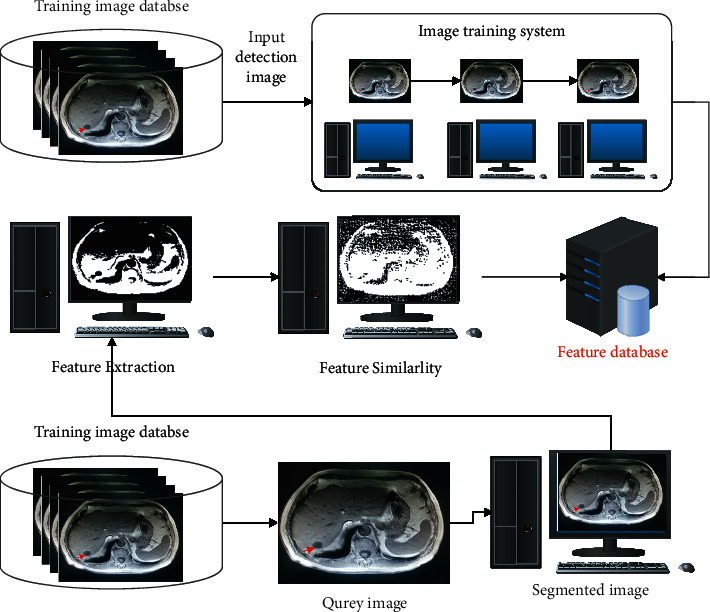
IAECP-based colorectal cancer prediction system architecture.

**Figure 2:**
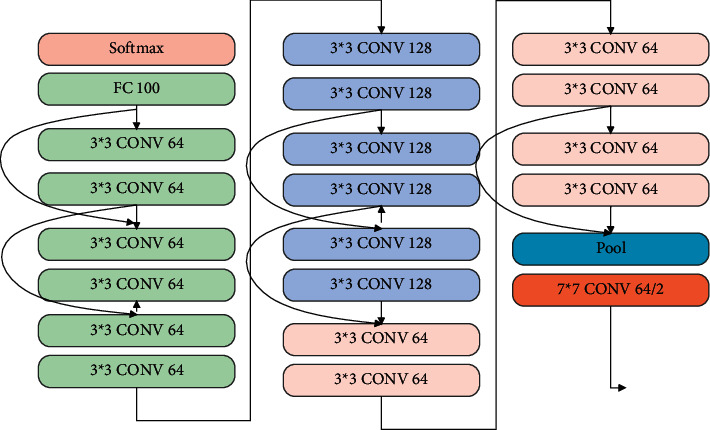
ResNet deep neural network block structure.

This manuscript introduces residual deep learning with the sum absolute cross-correlation template feature matching (SACC) algorithm for creating the IAECP system to predict colorectal cancer from the database. Here, Figure 1 represents the in-depth colorectal cancer detection framework using the introduced approach. For image segmentation, feature extraction, and feature training process in this work, the ResNet deep learning system is used on the histological image data set [18] (contains more than 100,000 images). Sum absolute cross-correlation template feature matching (SACC) algorithm analyzes the similarity between the derived features and the template features for predicting the colorectal cancer features. Here, ResNet deep neural [19] network is used to extract the features from the colorectal cancer image, which is processed using the SACC. Template matching is an approach in digital image processing for identifying small parts of an image that match a template image. Combining template matching methods such as dice coefficient and normalized cross-correlation with a robust algorithm yields a significant advancement in the accuracy ratio for cancer detection and recognition. The ResNet structure is depicted in Figure 2. Here, the ResNet, a deep learning system, uses the residual function, which examines the predicted actual value for extracting the features based on the training process. This residual value minimizes the deviation between the values that are computed in every layer of the network. Hence, the ResNet deep learning method works on the histological image data set for examining the polyps located in the image in an improved manner. The effective feature learning and activation function in the ResNet network process utilizes a high level of features that reduces manual cancer detection errors. Then the elaborate working process of the IAECP colorectal cancer detection process is explained in the below subsection.

Figure 1 represents the residual deep learning with the sum absolute cross-correlation template feature matching (SACC) algorithm-based colorectal cancer prediction process. During this process, the residual deep learning process is used for the feature training process, which minimizes the deviation between the predicted and actual value. The query images are examined; image regions are extracted along with the edges. From the derived edge image, various features are extracted and matched with the database using SACC. During the feature residual feature training process, the utilized neural network layers are processed as represented in Figure 2.

The ResNet consists of several blocks of layers [20], where each specific convolution functions for processing the input colorectal cancer images. To solve the vanishing/exploding gradient problem, this architecture introduced the concept called residual network. Because neural networks are good function approximators, the identification function should easily be solved in which the output of a function is the entry itself. According to the same reasoning, the network should anticipate which function it had previously learned by additional input when we provide the input to the first level of the model to be the output of the last layer of the model. With ResNets, the gradients can flow from later levels to initial filters straight through the skip connections.

### 3.1. Data Set

**Figure 3:**
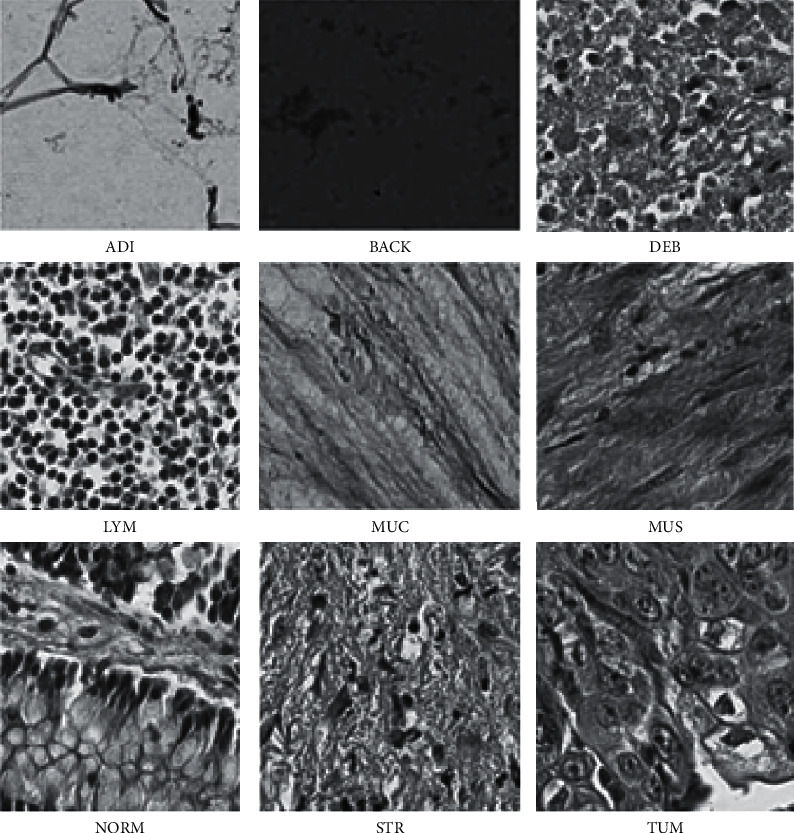
100,000 histological colorectal cancer image data set sample images.

In this research work, a publicly available data set called 100,000 histological colorectal images [21] is used to identify cancer (available at https://zenodo.org/record/1214456#__sid = js0). The data set consists of 100,000 nonoverlapping hematoxylin and eosin image patches. Along with this, stained histological images from colorectal cancer patients and normal tissue information available, which has been taken for analysis. These collected images have 224 ∗ 224 image pixels at 0.5 microns per pixel. The colorectal cancer images have different classes such as background (BACK), adipose (ADI), debris (DEB), smooth muscle (MUS), lymphocytes (LYM), mucus (MUC), colorectal adenocarcinoma epithelium (TUM), cancer-associated stroma (STR), and normal colon mucosa (NORM). These collected colorectal images are color-normalized by applying Macenoko's method. According to this collected colorectal cancer, sample images are depicted in Figure 3.

**Figure 4:**
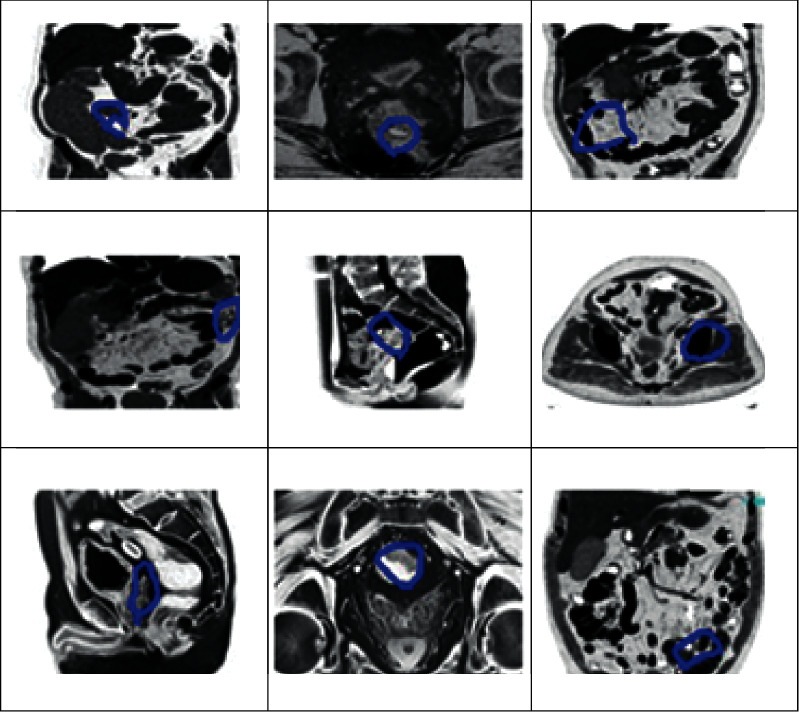
Sample colorectal cancer MRI images.

With the help of these color colorectal cancer images, respective MRI images are collected, which are processed using an effective deep learning system for predicting colorectal cancer. Then the related colorectal cancer images are shown in Figure 4.

In this work, 84,000 images are used for training purposes; 7,180 images collected from 50 colorectal adenocarcinoma patients are used for image validation purposes; and the remaining 8,820 images are used for testing purposes. The NCT tissue bank provides these histological colorectal cancer tissue samples.

### 3.2. Preprocessing

A total of 100,000 histological colorectal cancer images of 224 ∗ 224 tissue-related MRI images are directly applied to the residual deep learning network. The deep learning system uses the kernel value to the pixel intensity. The intensity value does not analyze the same or fixed in the MR image, varied according to the subjects. During the colorectal cancer recognition process, the data mining technique needs to be applied to preprocess the colorectal image. The normalization process has the same series of input images while removing noise from the image. Moreover, this is very helpful to process the image weight and bias value stably. So the colorectal images are normalized [22] according to the pixel intensity. For this purpose, the min-max normalization process is applied to eliminate the unwanted pixels in the image.

(1) $$\begin{array}{c} {\text{y}}_{\text{i}}=\frac{\left({\text{x}}_{\text{i}}-\min\left(\text{x}\right)\right)}{\left(\max\left(\text{x}\right)-\text{min}\left(\text{x}\right)\right)}. \end{array}$$

where normalized image value is denoted as y_i_, max(x) is the maximum intensity value of the image, and min (*x*) is the minimum value. This process normalizes the image, which is processed by ResNet deep learning system.

### 3.3. Image Segmentation

**Figure 5:**
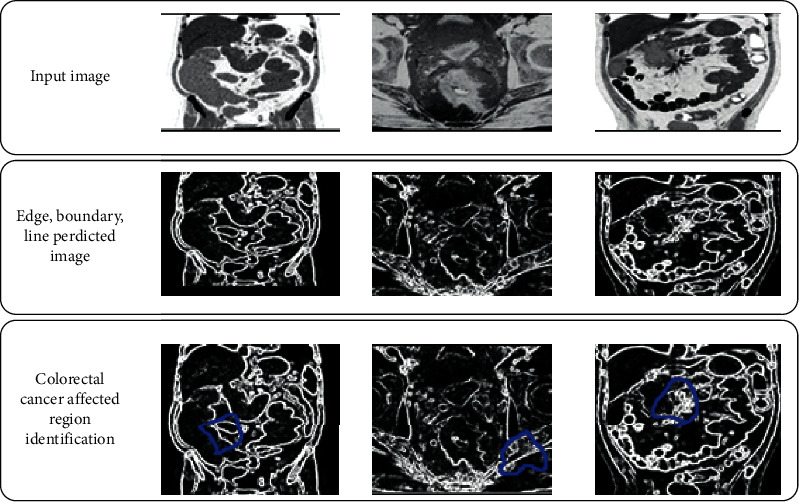
Deep-learning-based region segmented image.

The next important step is image segmentation, in which images are divided into different subimages. The divided images are more useful while extracting colorectal cancer-related features. At the time-image segmentation [23], lines, edges, curves, and other boundary information are getting from the MRI image. In this manuscript, a fully convolution deep learning method extracts the affected region from the colorectal image. The network takes the preprocessed MRI image as input. The full convolution network have several layers; each layer performs a specific function; and the image boundaries are extracted by mapping the pixels in the image. In this segmentation process VGG-16 semantic segmentation [24] process is used in which 1 × 1 convolution processes are used. During this process, transposed convolutions are applied for upsampling the low-resolution related pixel value. After that, VGG_16 lower layers are applied to map the high-resolution image features in the region. According to this process, the FCN-8 convolution network is used to segment the region from the colorectal cancer image. According to this discussion, the respective regions are segmented as shown in Figure 5.

### 3.4. Feature Extraction

The next step is feature extraction that is done with the help of a deep learning approach [25]. The deep learning system effectively extracts the feature from the image automatically. Moreover, the deep learning system derives the high- and low-level features from the image successfully. Due to effective derivation of the features from the image, which has been reduced based on the difficulties while classifying the query image features. The feature extraction process starts from the first input layer to the final layer in the convolution network. The most significant characteristic of these high data sets is that they have a huge number of variables. Feature extraction supports getting the best feature from those large data sets by choosing and combining variables into features, thereby efficiently decreasing the amount of data. Like the feedforward neural network, an error has to be backpropagated while deriving the features from the image, in which, first layer neuron *i* gets the input from the l-1 layer in *j* neuron.

(2) $$\begin{array}{c} {\text{In}}_{\text{i}}^{l}=\sum_{\text{j}=1}^{\text{n}}{\text{W}}_{\text{ij}}^{\text{l}}{\text{x}}_{\text{j}}+{\text{b}}_{\text{i}}. \end{array}$$

where *w* is the weight value of the network, *b* is the bias value, and *i* and *j* are input neurons in layer *l*. Here, the entire neurons are fully connected and convolutional layers. Hence, the output is computed using (2) and (3). Furthermore, the following formula is calculated:

(3) $$\begin{array}{c} {\text{out}}_{\text{i}}^{l}=\text{max}\left(0,{\text{In}}_{\text{i}}^{l}\right). \end{array}$$

The output of each layer is obtained in the form of non-linear activation. Then the mapping process is formed to get the image features done using the pooling layer [26]. The pooling layer has a k ∗ k square window sliding with an N ∗ N feature map. The layer takes the mean value of features that minimize the feature map spatial size inside the window. The reduced spatial size is represented as follows:

(4) $$\begin{array}{c} \text{N}*\text{N to}\frac{\text{N}}{K}*\frac{\text{N}}{K}. \end{array}$$

The spatial size gets from a single identified region (K ∗ K) in the colorectal image. At last, each pixel feature value is computed in the final layer using the softmax function computed using the following formula:

(5) $$\begin{array}{c} {\text{out}}_{\text{i}}^{l}=\frac{{\text{e}}^{{\text{In}}_{\text{i}}^{l}}}{\sum_{\text{i}}{\text{out}}_{\text{k}}^{l}}. \end{array}$$

According to the weight value, the network deviation and cost function are minimized during the feature extraction process estimated using the following formula:

(6) $$\begin{array}{c} \text{C}=-\frac{1}{\text{m}}\sum_{\text{i}}^{m}\text{ln}\left(\text{p}\left({\text{y}}^{\text{i}}|{\text{X}}^{\text{i}}\right)\right). \end{array}$$

where the total number of features in the feature set is denoted as *m*; furthermore, *i*-th feature in the set is represented as X^i^ andy^i^ that are the output value, and the true feature classification probability value is denoted as (p(y^i^*|*X^i^)). Here, stochastic gradient descent function is utilized to reduce the cost function while extracting the features. Furthermore, *W* is the weight value of the *t* iteration of *l* convolution layer [27]. As discussed earlier, the error rate is reduced by updating the weight value that is done as follows:

(7) $$\begin{array}{c} \gamma^{t}=\gamma^{tN/m}. \end{array}$$

where *γ*  is scheduling rate, *m* is the feature set, *t* is iteration, and *N* represents features. The weight updating process is defined as follows:

(8) $$\begin{array}{c} V_{l}^{t+1}=\mu V_{l}^{t}-\gamma^{t}\alpha_{l}\frac{\partial \hat{C}}{\partial W_{l}}, \end{array}$$

(9) $$\begin{array}{c} W_{l}^{t+1}=W_{l}^{t}+V_{l}^{t+1}. \end{array}$$

where *α*_l_ is the learning rate of *l* layer. Momentum is denoted as *μ*.

According to this process, the features are derived from providing proper training to the image features. The training is done by applying the ResNet deep neural network.

### 3.5. ResNet Deep Neural Network Feature Training

The important step is feature training; in this work, the residual deep neural network is used. This network establishes the training process according to the cerebral cortex. Moreover, ResNet uses skip connections over some layer concepts to train the features. Hence, the feature training process was developed by skipping the double or triple layer of batch normalization and non-linearities. Along with this additional weight, values are used to skip the weight named the Highway Nets. During the training process system, a more parallel skip is named from the DenseNet. The training process uses the different layers in which the predicted value is compared with the actual value using the residual function. As discussed earlier, the network uses skip connections; hence, layers of single skip are denoted as *l* to *l*+2 pr l-2 to *l*. These representations are denoted as the indexing system, which helps identify the forward or backward. For a single skip, the single flow in the forward layer is represented as *l* + K, which uses the backpropagation rule while training the feature set. Here, K is denoted as the skip number. In addition to this layer skip, a layer has the weight value that is denoted as W^l−1,l^ in layer l-1 to *l*. From layer l-2 to *l*, system uses the W^l−2,l^. After initializing the layers and respective weights, input forward propagation is computed as follows:

(10) $$\begin{array}{c} {\text{a}}^{\text{l}}=\text{g}\left({\text{W}}^{\text{l}-1,\text{l}}.{\text{a}}^{\text{l}-1}+{\text{b}}^{\text{l}}+{\text{W}}^{\text{l}-2,\text{l}}.{\text{a}}^{\text{l}-2}\right), \end{array}$$

(11) $$\begin{array}{c} {\text{a}}^{\text{l}}=\text{g}\left({\text{Z}}^{\text{l}}+{\text{W}}^{\text{l}-2,\text{l}}.{\text{a}}^{\text{l}-2}\right). \end{array}$$

where *l* layer activation function is denoted as a^l^, *l* layer activation function is represented as *g*, and weight matrix is W^l−1,l^ in layer l-1 to *l*.

(12) $$\begin{array}{c} {\text{Z}}^{\text{l}}={\text{W}}^{\text{l}-1,\text{l}}.{\text{a}}^{\text{l}-1}+{\text{b}}^{\text{l}}. \end{array}$$

During the ResNet forward propagation process, the explicit weight matrix is denoted as W^l−2,l^, and the respective activation function is simplified as follows:

(13) $$\begin{array}{c} {\text{a}}^{\text{l}}=\text{g}\left({\text{Z}}^{\text{l}}+{\text{a}}^{\text{l}-2}\right). \end{array}$$

So, in general, ResNet-based input forward propagation is denoted as follows:

(14) $$\begin{array}{c} {\text{a}}^{\text{l}}=\text{g}\left({\text{Z}}^{\text{l}}+\sum_{\text{k}=2}^{\text{K}}{\text{W}}^{\text{l}-\text{k},\text{l}}.{\text{a}}^{\text{l}-\text{k}}\right). \end{array}$$

**Figure 6:**
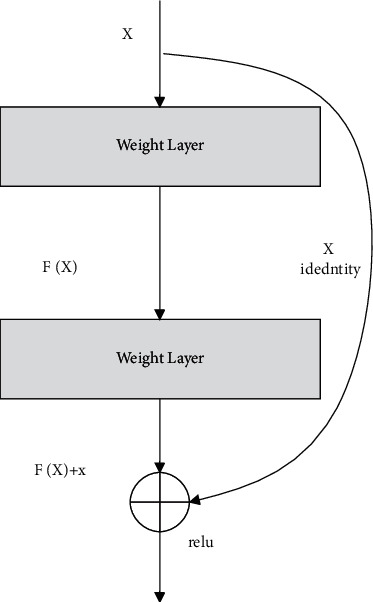
ResNet block structure.

In (12)–(14), the identity matrix should be formulated for W^l−2,l^ when the dimension is matched. The identity block is created when the activation function is passed from l-2 to *l* without weighting value. According to the discussion, the ResNet forward propagation-based feature training process is depicted in Figure 6.

After computing the input-related output value, if the network has an error value, that must be backpropagated to the network for predicting the exact output value. Hence, the backpropagation learning is performed as follows:

(15) $$\begin{array}{c} \Delta W^{l-1,l}=-\eta \frac{\partial E^{l}}{\partial W^{l-1,l}}=-\eta a^{l-1,l}.\delta^{l}. \end{array}$$

(15) is computed to the normal path, and the propagation value is estimated when a network have the skip path as follows:

(16) $$\begin{array}{c} \Delta W^{l-2,l}=-\eta \frac{\partial E^{l}}{\partial W^{l-2,l}}=-\eta a^{l-2,l}.\delta^{l}. \end{array}$$

where the learning rate is *η*, layer *l* error signal is denoted as *δ*^*l*^, and *l* layer activation function is *a*_*i*_^*l*^. So, in general, the weight matrix for the K skip path is computed using the following formula:

(17) $$\begin{array}{c} \Delta W^{l-k,l}=-\eta \frac{\partial E^{l}}{\partial W^{l-k,l}}=-\eta a^{l-k}.\delta^{l}. \end{array}$$

According to the learning rule, the weight matrix is merged, and the same process is performed in the learning process. This process is repeated to train the colorectal cancer-related information and stored in the database. When the new incoming features are entered into the system, the feature matching process is performed with trained and query image features.

### 3.6. Feature Matching Using Sum Absolute Cross-Correlation Template Feature Matching (SACC)

The final step of this IAECP system is feature matching is performed by applying the sum absolute cross-correlation template feature matching (SACC) algorithm. The feature matching process [28] examines the specific image features used to predict colorectal-cancer-related images. The computed output value is highest when the query image structure is matched with the trained image feature value. Here, the query image is denoted as S(x,y), in which *x* and *y* are denoted as the query image pixel values. The query image is compared with the template image T(x_t_, y_t_) in which x_t_, y_t_ is represented as the template image pixel points. After making the initialization, the template image origin value with the query image S(x,y) should estimate the sum of the product between the image coefficients S(x, y) andT(x_t_, y_t_). As discussed above, the query image S(x, y) coordinates (X_s_, Y_x_) that has the intensity value I_s_(X_s_, Y_s_). Likewise, the template image has the intensity value that is denoted as I_t_(X_t_, Y_t_). Then the absolute difference between the pixel intensity must be computed as follows:

(18) $$\begin{array}{c} \text{Difference}\left({\text{X}}_{\text{s}},{\text{Y}}_{\text{s}},{\text{X}}_{\text{t}},{\text{Y}}_{\text{t}}\right)=\left|{\text{I}}_{\text{s}}\left({\text{X}}_{\text{s}},{\text{Y}}_{\text{s}}\right)-{\text{I}}_{\text{t}}\left({\text{X}}_{\text{t}},{\text{Y}}_{\text{t}}\right)\right|. \end{array}$$

After computing the pixel intensity difference, the sum of absolute difference (SAD) should be estimated using the following formula:

(19) $$\begin{array}{c} \text{SAD}\left(\text{x},\text{y}\right)=\sum_{\text{i}=0}^{{\text{T}}_{\text{rows}}}\sum_{\text{j}=0}^{{\text{T}}_{\text{cols}}}\text{Difference}\left(\text{x}+\text{i},\text{y}+\text{i},\text{i},\text{j}\right). \end{array}$$

Then the computed origin value SAD is computed the entire image feature value that is calculated using the following formula:

(20) $$\begin{array}{c} \sum_{\text{x}=0}^{{\text{S}}_{\text{rows}}}\sum_{\text{y}=0}^{{\text{S}}_{\text{cols}}}\text{SAD}\left(\text{x},\text{y}\right). \end{array}$$

where S_rows_ represents the row-wise feature details, and column-wise features of image are denoted as S_cols_ of search image. This process is repeated continuously to predict the colorectal cancer image effectively. Thus, the introduced deep learning system successfully trains the feature extracts. Due to the extracted high-level feature information, the system matches the template and query images effectively. Then the efficiency of the system is evaluated using experimental results and discussion.

## 4. Experimental Results and Discussion

To evaluate the efficiency of the introduced IAECP system, it uses 100,000 histological colorectal images. The data set consists of several images, and those images are divided for testing, training, and validation purposes. Among the several images, 8,820 images are used for testing purposes. During this process, a fivefold cross-validation approach is used for examining the efficiency of the system. Here, collected colorectal cancer images are divided into *k* equal samples, in which one part is used for testing, one part is used for validation purposes, and the remaining parts of histological images are used for training purposes. The test images are treated as a query image compared with the trained images to predict colorectal cancer images. The discussed IAECP colorectal cancer predicted images are developed using the MATLAB R2017b tool.

### 4.1. ResNet Training Process

**Figure 7:**
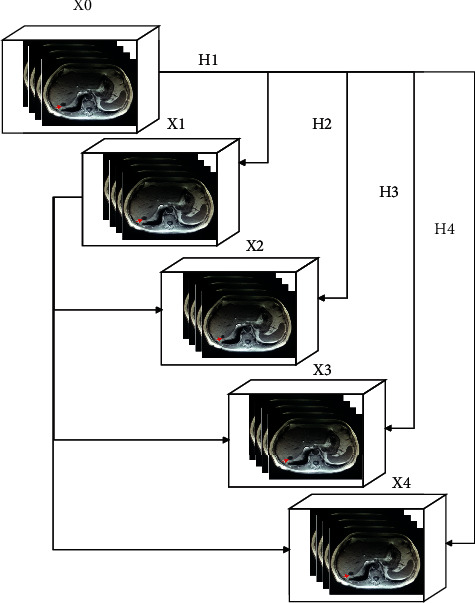
ResNet deep neural network training process structure.

The colorectal cancer image training process using ResNet deep learning system consumes nearly 20 to 30 minutes; it varies according to the data set's size, convergence, and parameter size. To achieve the highest colorectal cancer recognition accuracy, the feature training should be performed perfectly because the trained features are played an important role in the testing process. Then the structure of the ResNet deep learning system training process is depicted in Figure 7. The ResNet has several blocks; the block uses the input with 28 ∗ 28 size that uses the 1 generator multiplier while initiating the training process. The inputs are transmitted to the convolution layer that out maps are 64, kernel size is (5 ∗ 5), padding (4 ∗ 44), strides (1 ∗ 1), dilation (1 ∗ 1), and 1,600 cost parameters are used. These convolution parameters are successfully processed the 28 ∗ 28 size input image and produce the 64 ∗ 32 ∗ 32 output image. After that, the obtained output is transmitted to the batch normalization process, in which a 0.5 decay rate and 256 cost value are used, and the 64 ∗ 32 ∗ 32 output value is obtained. The batch normalized values are fed into the ReLU unit in which output is produced as same as the output value 64 ∗ 32 ∗ 32. This ReLU unit output value is fed into the next block 2 convolution process, which uses the 64 out maps, kernel (1 ∗ 1), padding (0 ∗ 0), strides (1 ∗ 1), dilation (1 ∗ 1), and 4,096 cost parameters to get the 64 ∗ 32 ∗ 32 output value. The obtained output is processed in the batch normalization process using a 0.5 decay rate and 256 cost parameter and the 64 ∗ 32 ∗ 32 output value. Likewise, ReLu-2, convolution 3 (kernel shape (3 ∗ 3)), batch normalization, ReLU-3, convolution kernel shape (1 ∗ 1), convolution 4 (kernel shape (1 ∗ 1)) and batch normalization 4 are used to get the 64 ∗ 32 ∗ 32 output value. Then the max-pooling process should be performed to map the features while training the feature set. The max-pooling process uses the 64 ∗ 32 ∗ 32 input image, (2 ∗ 2) kernel shape, (2 ∗ 3) strides, (0 ∗ 0) padding and 65,536 cost parameters that produce the 64 ∗ 16 ∗ 16 output image. Then the above process is repeated continuously until it reaches 64 ∗ 4 ∗ 4. Then average pooling process is performed to get the 64 ∗ 1 ∗ 1 output value that utilizes the (4 ∗ 4) shape. Finally, the affine parameter is applied to get the output in the network layer. During the training process, the softmax activation function is applied to know the exact class value of colorectal cancer features. This training process mostly helps increase overall cancer recognition accuracy.

**Figure 8:**
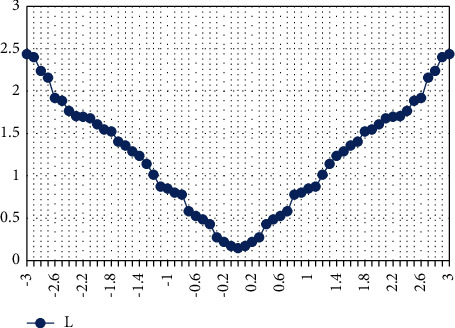
ResNet-DLS–loss value representation.

This training process minimizes the variation between the testing and training variables. The ResNet DLS system reduces the error rate values such as the mean square error rate during the computation process. At that time, the network reduces the Huber loss from the query variable and the data set variable, successfully resolving the regression problem. The obtained Huber loss value while examining the colorectal cancer images is depicted in Figure 8.

**Figure 9:**
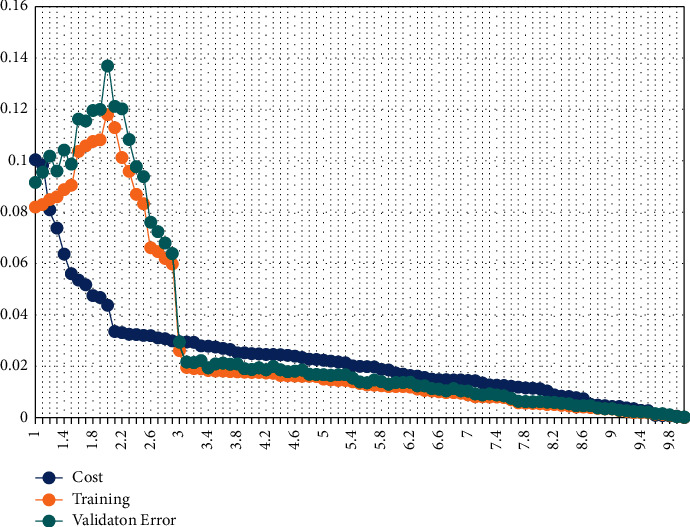
ResNet learning curve representation.

In Figure 8, *i* represents the difference between the query image variable and trained data set variable, and *δ* is the threshold value of the network. The ResNet training process has a minimum error or loss value while training the features from the analysis. In (18) and (19), the difference between the query image variable and the trained data set variable has been computed. With the help of the training process, the obtained learning curve for training and validation error value is depicted in Figure 9.

Figure 9 depicted that the system ensures a minimum error rate on a different number of epochs. As discussed earlier, the training efficiency only determines the overall colorectal cancer recognition accuracy. Hence, the training process is validated, and the obtained result is shown in Table 1.

With the help of the training process efficiency, the overall colorectal cancer recognition system proficiency is determined using the following performance metrics.

### 4.2. Performance Metrics

N image features are stored in the database, which compares the query image with the training image. The comparison between these trained with query images provides the colorectal cancer details. The efficiency of the system is evaluated using precision, recall, and accuracy metrics.

(21) $$\begin{array}{c} \text{precision}=\overset{\text{k}}{\underset{\text{j}=1}{\sum }}\frac{{\text{T}}_{\text{j}}}{\text{K}}, \end{array}$$

(22) $$\begin{array}{c} \text{recall}=\sum_{\text{j}=1}^{\text{k}}\frac{{\text{T}}_{\text{j}}}{\sum_{\text{j}=1}^{\text{N}}{\text{T}}_{\text{j}}}. \end{array}$$

where K is the number of features derived from the database, Tj is the relevance between the two image features, xj is the query image, and xi is the trained image. The matching between xi and xj is equal then the Tj having the value 1 otherwise 0. In addition to this, the accuracy metric is calculated using the following formulas:

(23) $$\begin{array}{c} \text{accuracy }=\left(\text{TP}+\text{TN}\right)/\left(\text{TP}+\text{TN}+\text{FP}+\text{FN}\right)*100\text{\% } \end{array}$$

(24) $$\begin{array}{c} \text{F}1-\text{score}=2.\frac{\text{precision}.\text{recall}}{\text{precision}+\text{recall}}. \end{array}$$

Using these performance metrics, the excellence of the IAECP-based colorectal cancer prediction system efficiency is evaluated.

### 4.3. Results of the Proposed Method

Here, the efficiency of ResNet DLS with SACC algorithm efficiency is evaluated. As discussed earlier, the fivefold cross-validation process uses the excellence of the system. During the training process, different block layers are used to examine the system. From our training parameters, the efficiency of the system improved gradually. In addition to this, a deep learning system is used in the image segmentation and feature extraction process. These processes derive detailed and high-level information from the colorectal image effectively.  Table 2 depicted the colorectal tumor retrieval performance of various cancer types.

From Table 2, the IAECP system retrieves the different types of colorectal cancers effectively. Five types of colorectal cancer features are successfully derived using the fivefold cross-validation part. Furthermore, the system's excellence is evaluated using the testing image features, and the obtained results are depicted in Table 3.

From Table 3 it depicted that the introduced ResNet deep learning system recognizes colorectal cancer with a maximum of 99.80% accuracy on set 4. Not only that, the IAECP system predicts cancer with high accuracy on the entire five data sets. This maximum recognition accuracy is attained due to the effective feature training process with the minimum deviation between the query and testing image features. In addition to this comparison process, the excellence of the system is compared with the existing classifier. The obtained results are depicted in Table 4.

**Figure 10:**
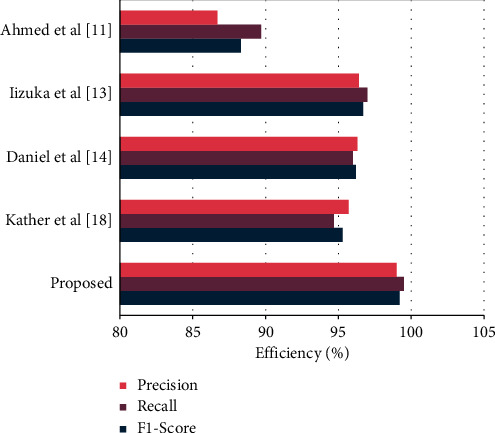
Comparison with existing research works.

Table 4 depicted that the introduced IAECP system can achieve 99.46% of accuracy while comparing the query image with the training image, 99.28% of accuracy while selecting the query image-related features from the database, and 99.65% of accuracy while choosing the specific query image features from the database. The data analysis clearly illustrated that the ResNet with DLS system and SACC matching algorithm ensures maximum accuracy compared to several research authors' methodologies. Then the relevant graphical representation is depicted in Figure 10.

### 4.4. Discussions

**Figure 11:**
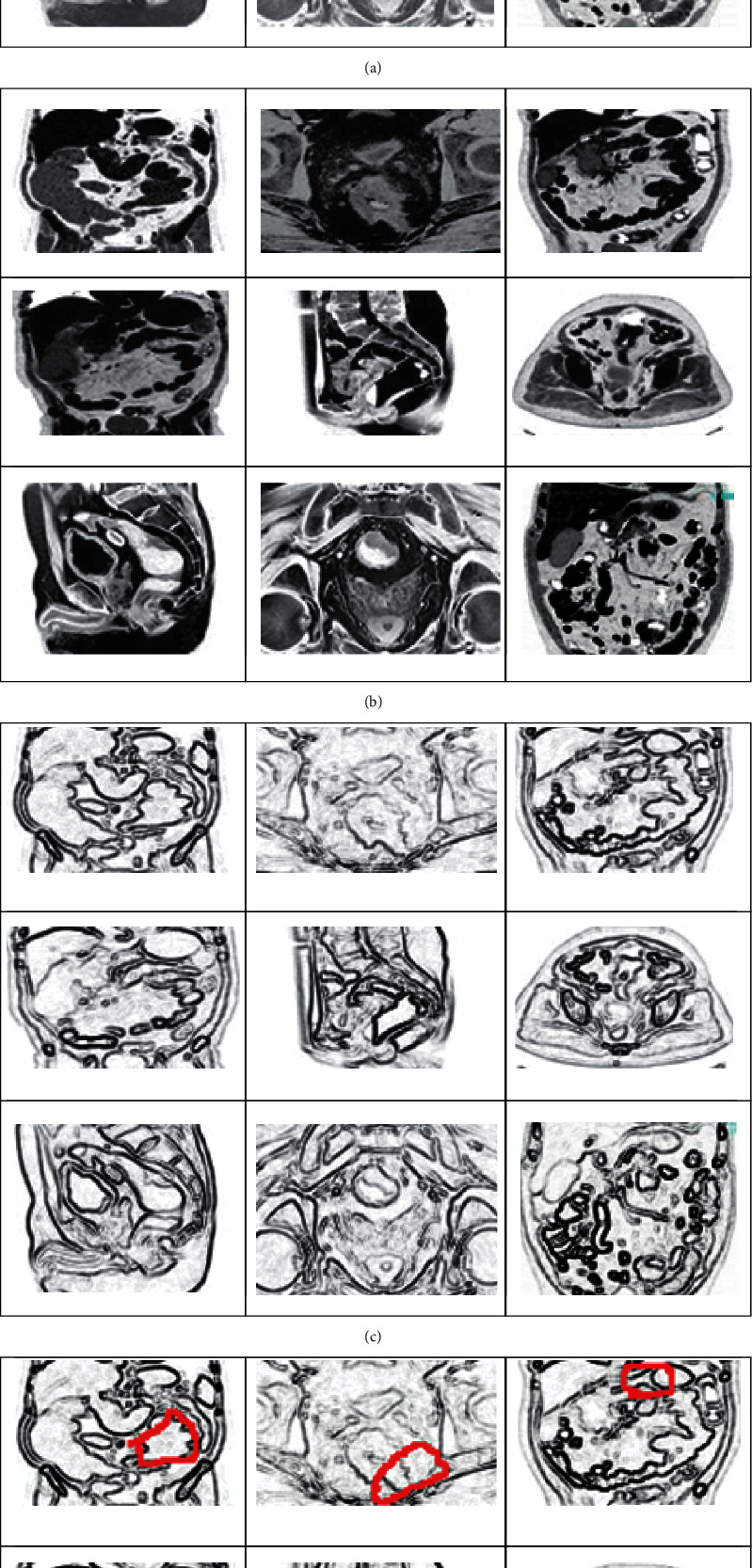
A deep learning system based on the colorectal cancer identification process step-by-step graphical representation: (a) colorectal MR noise removed the image; (b) quality enhanced colorectal MR image; (c) image boundary, edges, and line predicted colorectal images; and (d) colorectal-cancer-affected region identified images.

As per the above discussion, IAECP for the colorectal cancer detection system is created. The efficiency of the system depends on the ResNet deep-learning-based feature retrieval and feature training process. According to the excellent training process, the feature matching process is performed excellently. The similarity between the query features is compared according to the pixel intensity value of both trained and testing images. The next important factor is that multiple neural network layers perfectly analyze the image boundaries, lines, and edges. The successful extraction of this region identifies the colorectal cancer-affected region. From the derived region, different deep learned features are extracted automatically. These features are trained using multiple convolution layers, ReLu units, max-pooling layers, and batch normalization. These layers train the features without creating any complexity. These are the main reason to increase the IAECP colorectal cancer recognition process. According to the discussion, the system was evaluated using fivefold cross-validation process, in which the system ensures 99.80% of recognition accuracy. From the results, the cancer region segmented and feature detected images are shown in Figure 11.

**Figure 12:**
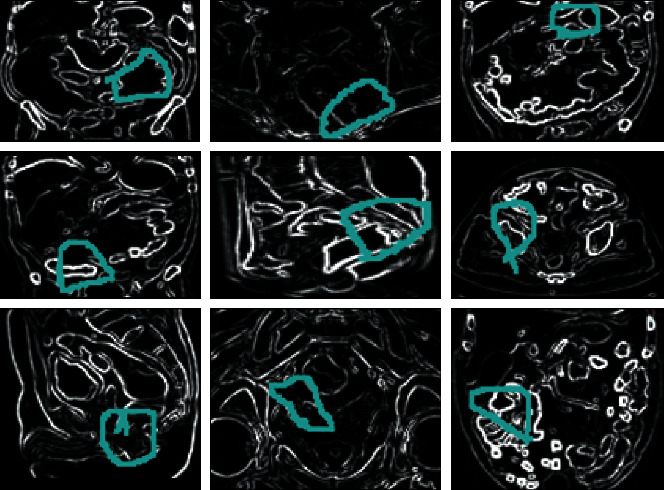
The visual representation of colorectal cancer-affected region features.

From the identified colorectal-cancer-affected region, different features are extracted using the ResNet deep learning network. According to the discussion in section 2, part D, the features are effectively derived from the colorectal MR image using multiple layers of convolution network. The derived features are depicted in Figure 12.

The extracted high-level features are mapped with the database to predict colorectal cancer. With the help of the features, the query image features are mapped pixel by pixel, and the exact location is identified successfully.

**Figure 13:**
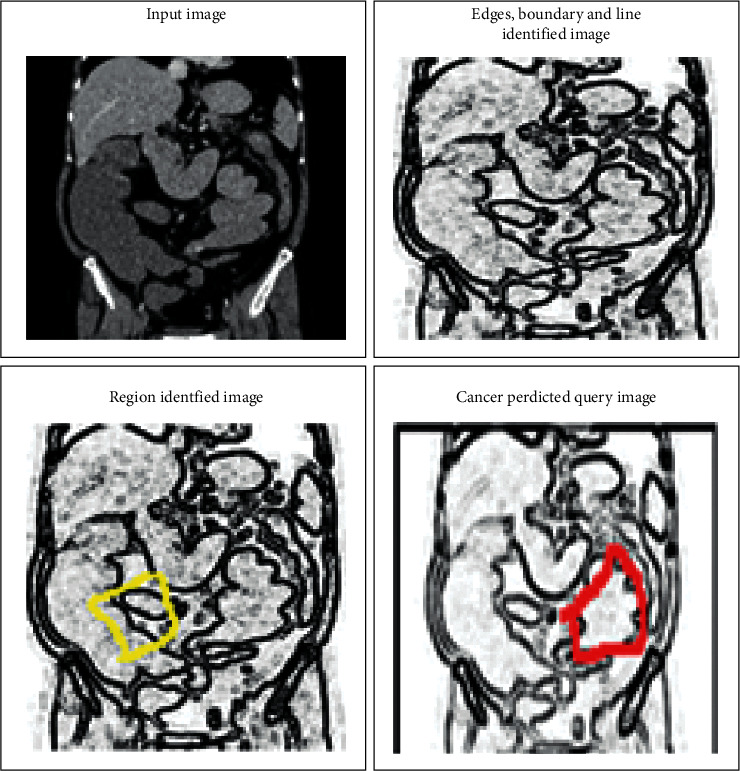
Colorectal cancer identification for query image.

Figure 13 depicted the results of the query image to predict colorectal cancer. The query image features are identified by comparing the query image details with the training image. From the comparison, the same feature representation and the information match with trained image one MR image. According to the existing representation, another cancer-affected region is detected successfully. Thus, colorectal cancer is predicted successfully with a high recognition rate (99.8%) and minimum deviation error achieved in both the training and testing process.

## 5. Conclusion

Thus, the paper creates the image analysis and earlier cancer prediction (IAECP) system to recognize colorectal cancer. In this work, 100,000 histological colon tissue images are collected from different patients. The relevant MRI images are used to analyze colorectal cancer. The image pixels are normalized by computing the minimum and maximum value, which makes computation easier. The affected region is identified by examining every pixel in the MR image. The various features are extracted from the image utilizing multiple layers of the convolution process. Residual deep learning networks train the extracted features. During the feature training process, the residual function computes the variation between the predicted and actual values. This computation process minimizes the deviation while comparing the training and query image. Given our approach to normal slides with excellent accuracy, algorithms in the neural network can help screen pathologists save time identifying tumor locations. This novel approach may be a great aid in the diagnosis of colorectal cancer. At last, the excellence of the system is evaluated using MATLAB tool-based results in which the system ensures 99.85% accuracy while predicting colorectal cancer. In the future, the system performance is enhanced by applying an optimized algorithm process.

## Figures and Tables

**Table 1:** ResNet DLS training efficiency.

Efficiency metrics	Efficiency
Accuracy	0.9924
Avg. precision	0.9926
Avg. recall	0.9923
Avg. F1-measure	0.9924

**Table 2:** Different cancer types recovery performance.

Cancer types	Precision	Recall	F1-score	
Adenocarcinoma	99.23	99.37		99.3
Gastrointestinal carcinoid tumor	99.48	99.38		99.43
Primary colorectal lymphoma	99.62	99.32		99.47
Gastrointestinal stromal tumor	99.38	99.18		99.28
Sarcoma	99.76	99.28		99.52

**Table 3:** Efficiency of the fivefold validation process.

Metric	Set 1	Set 2	Set 3	Set 4	Set 5
Precision	98.79	98.92	99.89	99.88	99.58
Recall	99.49	99.39	99.68	99.73	99.48
F1-score	99.1	99.15	99.785	99.805	99.53

**Table 4:** Comparison of existing classifiers with IAECP system for colorectal cancer recognition.

Classifiers	Precision (%)	Recall (%)	F1-score (%)
Ahmed et al. [11]	86.9	89.7	88.3
Iizuka, et al. [13]	96.39	96.89	96.64
Daniel et al. [14]	96.29	95.98	96.135
Kather et al. [18]	95.79	94.76	95.275
Proposed	99.28	99.65	99.465

## Data Availability

The data used to support the findings of this study are available from the corresponding author upon request.
